# Foreign Correspondence

**Published:** 1889-01

**Authors:** 


### Foreign Correspondence.

DR. W. XAVIER SUDDUTH,

Editor INTERNATIONAL DENTAL JOURNAL:

*Dear Sir:*—I am duly in receipt of your recent favor, and have given the contents careful consideration.

I quite believe there is room for an international journal of dental and oral surgery on the lines you indicate, and that edited by the gentlemen whose names you mention should be a success. I will be most happy to accept the appointment you so flatteringly offer me, and do what I can to further the interests of the new journal.

There are a few good men in Scotland whose contributions would do credit to any publication, and some of them I think I could move to active assistance.

But as regards this and other details, such as date of publication, price, etc., you will furnish me, with particulars, when the scheme is nearer maturity.

Knowing that you must at present have much correspondence to read and answer, I will not further trespass, but thank you and those associated with you for the confidence reposed in me.

I am, dear sir, yours truly,

W. BOWMAN MACLEOD,

16 George Square, Edinburgh, Scotland.

To the EDITOR:

Dr. Miller, with whom I am in correspondence with regard to dental matters, has kindly extended to me an invitation to act as a foreign correspondent to the "new journal."

The writing of elaborate papers is entirely out of my line, but I shall hope now and then to contribute little hints which seem to me to be of practical value; of which the following is one: In fitting crowns it is sometimes a difficult thing to decide just where to grind in order to form a perfect joint. The writer has found that by mixing, while melted, one part lampblack, by bulk, to five parts yellow or white wax, that an excellent material is formed for determining the point of contact.

The mass is perfectly opaque and shows accurately the point of articulation. The wax may be rolled into sticks when slightly cooled, or it may be poured, while melted, into glass tubes and allowed to cool. These tubes may afterwards be dipped into warm water and the "wax sticks" pushed out with the handle of an excavator. It is used in a manner similar to ordinary wax.

The crown and root are ground so as to fit approximately, then a small portion of the warmed wax is placed around the pin and the crown inserted. The points which come in contact will show accurately through the black wax and indicate where the grinding is to be done. The amount to be removed may be estimated by "sounding" the wax at the sides with a fine pointed instrument. The thickness of the wax will indicate just how much must be ground away to form a perfect joint. I know that the use of wax in this way is not new, but I have found the black wax so much better than the ordinary wax that I consider it of sufficient importance to tell of it.

I shall look with much interest on the first number of the new volume, and will take pleasure in adding my mite to help keep it up to its present high state of excellence. L. C. BRYAN,
Basle, Switzerland.

